# Supplementary material for: PAX1/JAM3 Methylation and HPV Viral Load in Women with Persistent HPV Infection
Source: Cancers (Basel). 2024 Apr 7;16(7):1430. doi: 10.3390/cancers16071430 (PMC11010937; doi:10.3390/cancers16071430)
Supplement: Supplementary file 1 [file cancers-16-01430-s001.zip › cancers-2896719-supplementary.pdf]

Supplementary Table S1

HPV distribution and correlation with PAX1^{m} / JAM3^{m} in 187 hrHPV genotypes

HPV genotype	Infection rate %(n)	Infection rate of HPV duration≥3 years,%(n)	PAX1^{m}		JAM3^{m}	
			Rho	*p*	Rho	*p*
HPV16	24.6(46)	16.6(15)	-0.252	0.090	-0.262	0.079
HPV52	23.5(44)	17.7(16)	-0.073	0.638	-0.023	0.884
HPV58	15.5(29)	10.0(9)	0.181	0.348	-0.085	0.661
HPV31	12.8(24)	7.8(7)	0.295	0.162	0.117	0.584
HPV56	10.7(20)	11.1(10)	0.167	0.480	-0.107	0.653
HPV39	9.6(18)	5.6(5)	-0.119	0.638	-0.108	0.668
HPV18	8.6(16)	7.8(7)	-0.226	0.398	-0.088	0.746
HPV51	7.0(13)	5.6(5)	-0.424	0.152	-0.582	0.040
HPV59	6.4(12)	4.4(4)	0.032	0.923	0.025	0.940
HPV66	6.4(12)	2.2(2)	0.483	0.115	-0.413	0.184
HPV33	5.3(10)	4.4(4)	-0.430	0.218	0.357	0.313
HPV68	3.7(7)	2.2(2)	0.393	0.396	0.464	0.302
HPV35	2.7(5)	3.3(3)	-0.400	0.517	-0.900	0.083
HPV45	0.5(1)	1.1(1)	-	-	-	-

Only one case of HPV45, the spearman correlation analysis was impossible.
